# An action plan for the agri-food sector at the time of the climate and biodiversity crises

**DOI:** 10.1093/nsr/nwad076

**Published:** 2023-03-17

**Authors:** Francesco Cherubini, Ting Hua, Wenwu Zhao

**Affiliations:** Industrial Ecology Programme, Department of Energy and Process Engineering, Norwegian University of Science and Technology (NTNU), Norway; State Key Laboratory of Earth Surface Processes and Resource Ecology, Faculty of Geographical Science, Beijing Normal University, China; Institute of Land Surface System and Sustainable Development, Faculty of Geographical Science, Beijing Normal University, China; State Key Laboratory of Earth Surface Processes and Resource Ecology, Faculty of Geographical Science, Beijing Normal University, China; Institute of Land Surface System and Sustainable Development, Faculty of Geographical Science, Beijing Normal University, China

## Abstract

Agriculture is responsible for about one third of global greenhouse gas emissions and it is the primary driver of habitat destruction. A paradigm shift embracing changes in lifestyles, agricultural practices, and policies is required to realize a sustainable transition in the agri-food sector.

Land areas on our planet are facing unprecedented levels of stress. More than 70% of the global ice-free land has been affected by human activities, 30% of the land is threatened by degradation, biodiversity and other ecosystem services (ESs) are declining, and climate change is altering ecosystem functioning [1]. Currently, agriculture is the dominant form of land use, with grazing land comprising 27% and cropland 12% (and more than half of the cropland is used to produce animal feed). Agriculture is responsible for about one-third of global greenhouse gas (GHG) emissions [2] and it is the primary driver of deforestation, habitat destruction, global freshwater withdrawals, and global ocean and freshwater pollution [3]. As land is a limited resource, it is subject to competition for food, water, health and other forms of well-being. The competition will be exacerbated by growth in population and affluence, unless significant changes in production and consumption patterns take place.

CO_2_ emissions from fossil fuel combustion are the main drivers of climate change, and reducing these emissions is essential for temperature stabilization. Many scenarios envision a complementary key role played by the agri-food sector that, while adapting to climate change, is expected to simultaneously mitigate its current emissions, contribute with negative emissions by sequestering CO_2_ into vegetation and soils, and reduce its land footprint to spare areas for production of renewable energy and biodiversity conservation. While some of these objectives can be co-delivered via nature-based solutions [4], others are mutually exclusive. For example, agroforestry can secure long-term yields of shaded crops while sequestering carbon in trees and soils and improving habitat quality, but there are trade-offs between high carbon storage and crop yields. Similarly, growing perennial grasses or trees on marginal land or within intensively managed agricultural landscapes can increase multifunctionality, soil organic carbon, and ESs, but achieving the large bioenergy or reforestation potentials expected by future scenarios implies conversion of large areas with consequent risks of impacting food security or water resources [1]. There is simply not enough land on our planet unless substantial changes in the agri-food system occur. Successfully tackling climate change, biodiversity decline and food security highly depends on effectively reducing the amount of land required to support our society and changing the way in which we manage land resources.

Multiple measures can be implemented from the supply and demand sides to reconcile food production with nature conservation and mitigation of climate change (Fig. 1). Among supply-side measures, regenerative farming methods (e.g. reduced tilling, crop rotation, etc.) can retain carbon and nutrients in soils and decrease the environmental impacts of agriculture. Optimal management of global cropland areas with the goal of closing current yield gaps can maintain current food production volumes using only half of today's cropland extent [5]. Shifting intensive monocultures into multifunctional systems, where conservation and carbon storage goals are actively integrated within agricultural landscapes, can generate large environmental benefits and improve human well-being. Additionally, effective allocation of land patches to nature conservation (e.g. natural revegetation or riparian buffers) can contribute to climate change mitigation, support species with small-range habitats, improve water regulation and reduce soil erosion [4].

**Figure 1.**
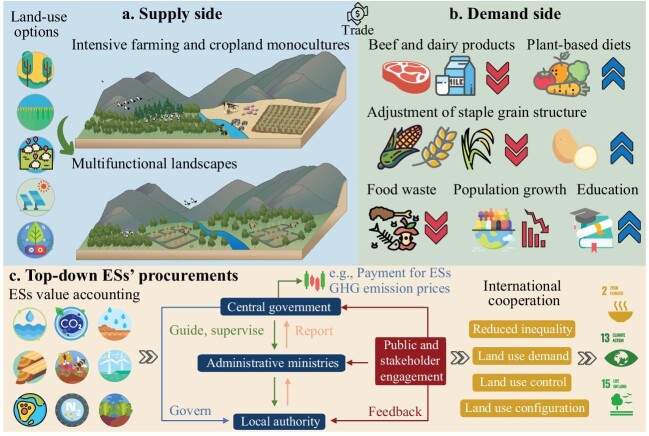
Supply-side and demand-side transformations for climate change mitigation and nature conservation, with the required policy framework aiming at promoting procurement of ecosystem services. More efficient production systems and closing existing yield gaps in developing countries can facilitate shifting intensive farming and cropland monocultures into multifunctional systems that reconcile food production with nature conservation and climate change mitigation (e.g. through options such as revegetation programs or other nature-based solutions, storing carbon in soils, or producing renewable energy) (a). Dietary changes aimed at reducing consumption of beef and dairy products, reducing food waste, increasing education (and food and sustainability literacy), controlling population growth, and/or adjustments in the staple grain structure of diets, can contribute to reducing pressure on land areas (b). An alternative governance system based on valuing ecosystem services and engaging central governments, local authorities and the public is essential to realize a sustainability transition in the land use sector (c), and secure a sustainable balance of food supply and demand via trade.

Among demand-side measures, dietary changes have the largest potential. Of the ∼4 billion hectares used today for agriculture, ∼80% are used for meat and dairy production. In an ideal scenario in which the world population substitutes all meat and dairy products with plant-based proteins, fish, chicken or pork, the global land used for agriculture would decrease by ∼75% (an area equal to North America plus Brazil) [3]. Natural revegetation of this land, and the corresponding reduction in emissions from food production, would achieve a mitigation of up to ∼500 GtCO_2_ by 2050 [6]. Controlling population growth and enhancing access to education are additional drivers to lower future food demand and promote more sustainable and healthy diets [7]. Transformations in the composition of staple foods by increasing the use of potatoes instead of maize, wheat or rice, would also decrease carbon emissions and water demand [8]. An additional potential lies in reducing food waste. About one-third of all the food in the world goes to waste, meaning that all the land, energy, emissions and water used in its life-cycle are wasted. Trade can favor a more sustainable agri-food system, for example by concentrating production on high-yield land. However, it can also have adverse effects. For instance, trade can stimulate expansion of production into high productive forested areas in countries where forest protection is weak, or market competition can accelerate degradation or pollution from intensive farming.

Despite some existing incentives to support a transition towards multifunctional landscapes that reduce environmental impacts of agriculture, most land policies are oriented towards productivity as they typically target national food security and incomes of rural areas [9].

As a result, agricultural incentives are mainly directed to farmers simply based on the area farmed, with little consideration of social or environmental benefits. Although approaches aimed at stimulating new financial frameworks based on ecosystem services are struggling to take off [9], the scientific community should continue to highlight their importance. It is increasingly clear that a global GHG emission pricing is necessary to bring land use emissions on track to become net negative by mid-century. Such pricing would help to prevent deforestation in developing countries, support carbon sequestration in managed areas, and reduce CO_2_ emissions from food production [7].

Integrating ESs into a GHG emission pricing scheme to promote more sustainable agriculture, coupled to incentives towards more plant-based diets, are key points of an action plan to guide sustainable land use by stimulating carbon sequestration into agricultural landscapes and reduce GHG emissions. In general, increasing carbon in vegetation and soils has positive cascading effects on multiple ESs, which in turn are connected to multiple sustainable development goals (SDGs). Provision of ESs, such as biodiversity or climate regulation, can be treated as public goods, and national governments are already committed to achieving SDGs 2, 13 and 15. Financial support can thus come from national or regional development funds and re-investing profits from global GHG emission pricing. To achieve this, a clear and transparent accounting and reporting system for ESs, inspired by current mechanisms that countries use to report GHG emissions, should be developed. Once countries compile a standardized accounting of ESs, ideally at a sub-national resolution, central governments can define high-level goals or priorities of land use and transfer the responsibilities for their achievements to administrative ministries and local authorities [10], which adjust action plans according to local threats, needs and public engagement.

Expanding international cooperation, public engagement, and international research on coupled human-natural systems is essential to identify and implement win-win solutions, but it will not prevent conflicts between (and among) global sustainability challenges (climate change mitigation, biodiversity conservation, or food security) and society. The transitions required for the achievement of environmental goals always come with social, economic and justice implications. Most land in use today, or manufacturing of food products, already delivers benefits to some stakeholders or consumers, and any intervention has consequences on the distribution of benefits. Because short-term trade-offs can penalize some actors, long-term benefits should be inclusive. Solutions should follow criteria that reduce inequalities, or use compensation mechanisms, financed through the new scheme discussed above.

Overall, a sustainable transition in the agri-food sector requires a paradigm shift that embraces both changes in lifestyles, agricultural practices and policies. Measures should be associated with inclusive socio-economic development and linked to the broader SDG agenda, or they risk exacerbating inequalities, especially in low- or middle-income countries where most land-based emission reductions and land conservation are expected to occur. As those countries have high economic, social and political limitations, overcoming global inequality and poverty alleviation go hand in hand with solving the climate and biodiversity crises. While securing the protection of natural areas, the current inefficiencies in land use warrant the promotion of land use changes and management that can reverse ongoing degradation processes and promote restoration of natural ecosystems. More land use change is beneficial in this context, and the required knowledge is largely available. Reducing the need for pastureland and expanding the presence of natural ecosystems and carbon storage within cropland landscapes are cornerstones to solving the biodiversity and climate crises. Their realization without harming food security and increasing inequalities is the moral duty our society is facing, from individuals to public and corporate levels.
